# 34 The Costs of Caring: Quantifying Compassion Fatigue and Compassion Satisfaction in Burn Therapists

**DOI:** 10.1093/jbcr/irae036.034

**Published:** 2024-04-17

**Authors:** Miranda L Yelvington, Tyler Corson, Jiale Hu, Rachel E Wood, Stacey Reynolds

**Affiliations:** Arkansas Children's Hospital, Little Rock, Arkansas; Virginia Commonwealth University, Ormond Beach, Florida; Department of Nurse Anesthesia/Virginia Commonwealth University, Richmond, Virginia; Virginia Commonwealth University, Richmond, Virginia; Arkansas Children's Hospital, Little Rock, Arkansas; Virginia Commonwealth University, Ormond Beach, Florida; Department of Nurse Anesthesia/Virginia Commonwealth University, Richmond, Virginia; Virginia Commonwealth University, Richmond, Virginia; Arkansas Children's Hospital, Little Rock, Arkansas; Virginia Commonwealth University, Ormond Beach, Florida; Department of Nurse Anesthesia/Virginia Commonwealth University, Richmond, Virginia; Virginia Commonwealth University, Richmond, Virginia; Arkansas Children's Hospital, Little Rock, Arkansas; Virginia Commonwealth University, Ormond Beach, Florida; Department of Nurse Anesthesia/Virginia Commonwealth University, Richmond, Virginia; Virginia Commonwealth University, Richmond, Virginia; Arkansas Children's Hospital, Little Rock, Arkansas; Virginia Commonwealth University, Ormond Beach, Florida; Department of Nurse Anesthesia/Virginia Commonwealth University, Richmond, Virginia; Virginia Commonwealth University, Richmond, Virginia

## Abstract

**Introduction:**

Recovery from a severe burn injury requires early and aggressive therapy, which is often painful and distressing to the patient. Burn therapists who guide these interventions may be prone to experiencing secondary trauma and compassion fatigue through repeated exposure to difficult situations. At the same time, therapists may gain a sense of purpose and altruism from their work, fostering compassion satisfaction. Despite being well documented in other health professions, compassion fatigue and compassion satisfaction have not been sufficiently explored among burn therapists. The purpose of this study was to quantify and characterize burn therapist’s compassion fatigue and satisfaction using the Professional Quality of Life (ProQOL) scale and to answer the question of what job-related factors may impact these outcomes.

**Methods:**

This cross-sectional study surveyed active burn therapists using Research Electronic Data Capture (REDCap). ProQOL scores were calculated for individual respondents and described for the sample using mean, standard deviation, and range. Multiple regression analyses were conducted to assess the impact of predictor variables of years in burn care, practice setting, and population on the subscales of the ProQOL for compassion satisfaction and compassion fatigue (burnout and secondary traumatic stress).

**Results:**

A total of 143 burn therapists were included, which exceeded the n needed to achieve statistical power. The included participants presented with low to moderate burnout scores (mean=23.5, range=11-38), secondary traumatic stress (22.5, 11-40), and moderate to high compassion satisfaction (40.4, 25-50). Regression analyses found years of practice significantly predicted (p ≤ .01) each of the compassion constructs.

**Conclusions:**

This study presents new knowledge for the field of burn care in its quantification of professional quality of life in burn therapists. While burn therapists experience moderate-to-high levels of compassion satisfaction in their work, burnout and stress also approached moderate levels. Years of practice was a significant predictor of compassion and may be related to increased competence or confidence in practice.

**Applicability of Research to Practice:**

This study is the first to study compassion fatigue and satisfaction in burn therapists. Understanding and addressing these issues are essential to recruiting and retaining a healthy and productive burn care workforce. Regular assessment of professional quality of life and career stage-specific supportive measures could provide a protective measure against compassion fatigue.

